# A Scoping Review on Fluorescence-Guided Surgery in Paediatric Renal Tumours: Current Perspectives and Future Plans

**DOI:** 10.3390/cancers18061041

**Published:** 2026-03-23

**Authors:** Max Pachl, Valerie Rudolf von Rohr

**Affiliations:** 1 Department of Paediatric Surgery and Urology, Birmingham Childrens Hospital, Birmingham B4 6NH, UK; 2 Institute of Cancer and Genomic Sciences, College of Medicine and Health, University of Birmingham, Birmingham B15 2TT, UK

**Keywords:** renal, cancer, near-infrared fluorescence, indocyanine green, B7H3, Glypican 3, GD2

## Abstract

**Background/Objectives**: Paediatric renal tumours, particularly Wilms tumours, have good survival outcomes following multimodal therapy; however, long-term morbidity related to nephrectomy and adjuvant treatment remains a major concern. As treatment paradigms increasingly prioritize nephron preservation and minimization of late effects, there is growing interest in technologies that can enhance intraoperative precision. **Methods**: A scoping review following the PRISMA guidelines was performed. We analysed articles on fluorescence for childhood renal tumours on 1 November 2025. Case reports, opinion articles, and narrative reviews were excluded. An Ovid Medline search with search terms “Kidney neoplasm” AND “Fluorescent Dyes”, along with a Cochrane trials registry search for “kidney” AND “neoplasm” AND “Fluorescent Dye”, was performed, along with a hand search of citations. **Results**: The Ovid Medline search yielded 21 results, and the Cochrane trials search gave 4 results. Following review, five papers were included, of which one was an ex vivo study and one was a randomised, controlled trial that is currently recruiting. **Conclusions**: There is a lack of evidence around the use of near-infrared fluorescence in paediatric renal tumour surgery. This review summarizes the key current findings and future perspectives.

## Simple Summary

Fluorescence-guided surgery using special dyes to identify specific tissues during surgery is growing in popularity. It can be used to identify lymph nodes that need removing to look for the spread of some cancers or tumours. Additionally, work is underway to develop dyes that show tumour borders, allowing surgeons to identify where cancers stop so they can remove all possible disease, as well as for the removal of less normal tissue. This review summarises recent work and future plans with respect to such dyes in paediatric renal cancer.

## 1. Introduction

Paediatric renal tumours account for approximately 5–7% of all childhood malignancies, and Wilms tumours (WTs) represent the most common renal cancer in children. Advances in multimodal therapy have led to improved overall survival, exceeding 90% in favourable-risk disease. Consequently, attention has increasingly shifted toward reducing treatment-related morbidity, particularly chronic kidney disease, hypertension, and cardiovascular sequelae, following nephrectomy and adjuvant therapy.

Surgical excision remains a cornerstone of treatment. In selected patients, nephron-sparing surgery (NSS) is now advocated to preserve renal function without compromising oncological outcomes [1]. However, accurate intraoperative delineation of tumour margins and identification of residual disease remain challenging, especially following preoperative chemotherapy or in minimally invasive approaches. Fluorescence-guided surgery (FGS) has emerged as a potential solution to these challenges by providing real-time, enhanced visualization of anatomic borders.

While extensively explored in adult oncological surgery [2,3,4], experience in paediatric renal tumours is limited but expanding, with Indocyanine Green (ICG) currently the only fluorescent dye licensed for use in children. ICG is widely used for perfusion assessment and lymph-node sampling; however, its optimal dose, administration strategy, and fluorescence intensity in paediatric tumour resection remain unclear. In selected tumours and patients, ICG can delineate tumour margins, and results from an ongoing randomised, controlled trial are anticipated to clarify its clinical utility in this population.

Cytalux, a folate receptor β-targeted fluorophore, is currently under evaluation in paediatric oncology trials in North America and represents a promising example of targeted fluorescence imaging [5,6]. In addition, conjugated fluorophores targeting GD2 receptors are being investigated in neuroblastoma [7,8]. Such approaches are well suited to many paediatric tumours, which are typically histologically homogeneous. In contrast, WTs demonstrate marked inter- and intratumoral heterogeneity both before and after neoadjuvant chemotherapy, with variable cellular markers and proteomic profiles [9]. This heterogeneity poses a major challenge for the development of effective conjugated fluorophores for primary, metastatic, or relapsed WTs, as any targeted agent would need to bind multiple tumour cell populations.

This review summarizes current applications of fluorescence-guided surgery (FGS) in paediatric renal tumour surgery, with a focus on ICG-based techniques; evaluates their strengths and limitations; and explores future directions, including tumour-specific fluorophores, ongoing clinical trials, and emerging conjugated agents.

## 2. Materials and Methods

### Study Design

**Figure 1:**
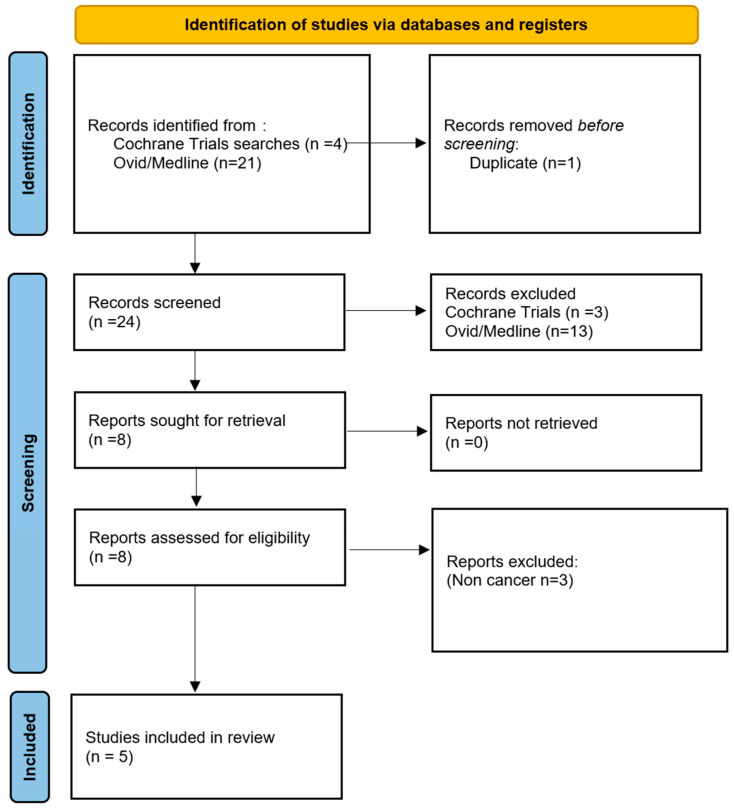
PRISMA workflow.

A scoping review of the medical literature and current/previous clinical trials relating to the use of fluorescence in paediatric renal tumours was conducted according to the Preferred Reporting Items for Systematic Reviews and Meta-analysis (PRISMA) guidelines (https://www.prisma-statement.org/) (Figure 1).

The search query [Supplementary File S1] was performed by a librarian trained in searches, along with the two investigators (M.P. and V.R.v.R.), using multiple electronic platforms, including PubMed, MEDLINE, Web of Science and Cochrane databases. The search included different combinations of terms and synonyms, such as “kidney neoplasms”, “Fluorescent Dyes”, “Fluorescent guided surgery”, “pediatric oncology” and “tumors”.

The inclusion criteria included articles in English and full-text articles describing the use of fluorescence-guided surgery in renal tumours in children from 0 to 18 years of age. Single case reports, systematic reviews (without meta-analysis), commentaries and conference communications were excluded.

For study selection, all databases were searched, and prior to screening, one study was excluded due to duplication. At screening (by M.P and V.R.v.R.), all studies were assessed for eligibility by review of titles and abstracts. Studies were excluded if they did not investigate a paediatric population, were not renal in nature, or did not investigate cancer and if they were review articles or case reports. Full texts were then reviewed, and studies were excluded if they were not related to renal tumours.

## 3. Results

Five papers were included in the review [10,11,12,13,14], of which two by Pachl et al. and Abdelhafeez et al. [10,13] exclusively dealt with tumour margins and two by Abdelhafeez et al. and Roberts et al. [12,14] exclusively dealt with nodes, whilst the last paper by Feng et al. looked at both tumour margins and nodes [11] (Table 1).

All papers concluded that ICG was safe and effective in its use following either intravenous, perihilar, or intraparenchymal injection.

**Figure 2:**
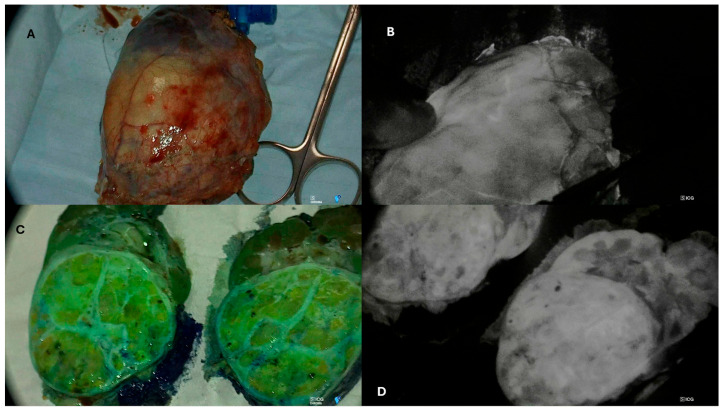
Ex vivo images of a paediatric blastemal Wilms tumour nephrectomy specimen under (**A**) white light and (**B**) near-infrared fluorescence. Ex vivo images of a bivalved nephrectomy specimen (**C**) in overlay mode and (**D**) under near-infrared fluorescence using Storz Endoskope Rubina NIR laparoscopes and a lens open-field camera (Karl Storz Endoskope, Tuttlingen, Germany).

Feng and Abdelhafeez assessed in vivo appearances of normal renal parenchyma and WTs following intravenous injection of ICG and concluded that following vascular washout, the dye remained in normal renal tissue but not in the tumour. Pachl agreed with this in an exploratory ex vivo study, its further work showed that blastemal disease was also hyperfluorescent and statistically the tumour to background appearance was similar to normal renal parenchyma. Therefore, although appealing, this technique appears to have limitations in WT surgery and may miss high-risk blastemal disease (Figure 2). As such, the ideal fluorescence marker is yet to be realised.

**Figure 3:**
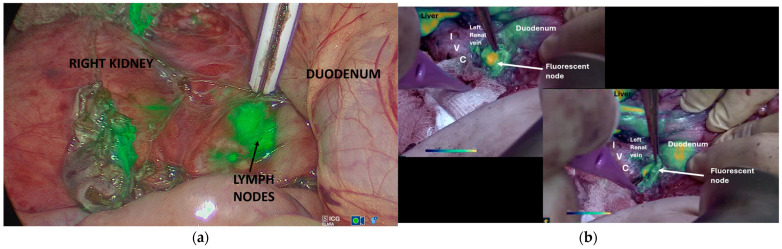
Intraoperative images of fluorescent lymph nodes during (**a**) minimally invasive (overlay mode) and (**b**) open radical nephrectomy for paediatric renal cancer (intensity mapping node) using Storz Endoskope Rubina NIR laparoscopes and a lens open-field camera (Karl Storz Endoskope, Tuttlingen, Germany).

For node identification, Roberts et al. showed that, in a small series, ICG Near-InfraRed Fluorescence (NIRF) was statistically more effective at increasing the nodal yield than standard white light alone. This is the only paper comparing NIRF and white-light groups. Additionally, this literature identified that nodes that were fluorescent could be histologically disease-positive or negative. This illustrates the lack of specificity of ICG for Wilms tumour deposits, although all patients had intermediate or low-risk disease as per the SIOP-RTSG guidelines [1]. However, limitations include the fact that the study was single-centre in nature and at risk of bias, and the number of patients was small; thus, the findings remain experimental. The authors concluded that the technique may be useful in identifying lymph nodes that the surgeon would not usually see and could provide a roadmap to nodal sampling (Figure 3).

No comparative or randomised, controlled studies have been published that identify whether ICG can improve nodal yield, although one is underway (GLO-Surgery ISRCTN26150156).

Based on these results, work is still needed in this field.

## 4. Discussion

Fluorescence is a physical property of certain molecules whereby the absorption of external energy causes electrons to transition from their ground electronic state to a higher-energy excited state. This excitation occurs when the molecule absorbs light of a specific wavelength. Once the excitation source is removed, the electrons rapidly return to their original energy level. During this relaxation process, a proportion of the absorbed energy is dissipated through non-radiative pathways as heat, while the remaining energy is released as light in the form of fluorescence. Because some energy is lost prior to emission, the emitted light has a longer wavelength and lower energy than the excitation light, a phenomenon known as the Stokes shift [15,16]. The emitted fluorescence can span a wide range of wavelengths, from the visible to the invisible regions of the electromagnetic spectrum.

Fluorescence-guided surgery exploits this principle in a manner analogous to the use of invisible ink: the fluorescent dye remains undetectable until illuminated with light at the appropriate excitation wavelength. Whereas invisible inks are typically excited by ultraviolet light, contemporary surgical fluorophores are excited by near-infrared (NIR) light, most commonly within the 750–820 nm range, which lies outside the visible spectrum [3,17]. NIR fluorescence is particularly well suited to surgical applications, as it permits greater tissue penetration, reduces tissue autofluorescence, and improves signal-to-noise ratios [4,18]. When excited by NIR light, the dye emits fluorescence that is detected by specialized camera systems and displayed as a real-time image overlay, allowing for visualization of anatomical structures, perfusion, lymphatic drainage, or tumour tissue that would otherwise be invisible to the surgeon [5,19].

The application of fluorescence in oncological surgery was first described in canine models, where Reynolds et al. demonstrated that indocyanine green (ICG) accumulated in spontaneous mammary tumours and their draining lymph nodes following intravenous administration [20]. Subsequent adult studies showed utility in identifying sentinel lymph nodes during lung [21] and breast cancer [2] surgery. With the increasing availability of near-infrared (NIR) imaging systems, interest expanded to tumour localization, including robotic renal tumour surgery, where differential fluorescence between normal renal parenchyma and tumour tissue was reported in 2011 [22]. Tumours were found to be variably hypo- or afluorescent, and these findings were later replicated in a retrospective review [23]. However, no reduction in positive surgical margins was observed compared with white-light surgery, although these results were limited by small sample sizes and potential selection bias.

Despite these limitations, fluorescence-guided surgery holds promise as an adjunct for renal tumour resection and lymph-node mapping. Surgical decision-making with respect to oncological procedures relies heavily on visual and tactile cues, yet tumour margins are often indistinct, increasing the risk of residual disease. Fluorescent imaging may provide additional optical guidance to improve margin identification. Furthermore, accurate sampling of draining lymph nodes is critical for staging, as nodal involvement frequently upstages disease and alters adjuvant therapy. Identifying relevant draining lymph nodes is challenging without tracers, and fluorescence-based techniques may facilitate more accurate and reproducible lymphatic mapping [24,25,26]. An overview of the most common current fluorophores is given in Table 2.

WTs are the most common type of renal malignancy in children. Management is guided by either the SIOP–RTSG UMBRELLA protocol [1] or the Children’s Oncology Group (COG) renal tumour protocols [27] and, for most patients, consists of multimodal therapy including chemotherapy and radical nephrectomy, with selected cases requiring radiotherapy (RT). Despite excellent overall survival rates, surgical technique remains a critical determinant of both oncological control and long-term renal function.

FGS has emerged as a potential adjunct to conventional surgical approaches, offering real-time visualization of tissue perfusion; lymphatic drainage; and, potentially, tumour margins. Its role in WT surgery is evolving, particularly in complex scenarios such as bilateral disease, where nephron preservation is paramount.

In unilateral WTs, radical nephrectomy remains the standard approach for most patients, with the primary aim of complete tumour excision and accurate staging through lymph-node sampling. In contrast, children presenting with bilateral synchronous WTs (SIOP stage V) undergo neoadjuvant chemotherapy followed by surgery. Surgical options include unilateral nephrectomy with contralateral nephron-sparing surgery (NSS); bilateral NSS; or, rarely, bilateral nephrectomy [28,29,30].

The objectives of surgery in bilateral disease are two-fold: to preserve as much functioning renal parenchyma as possible while achieving complete tumour resection with negative margins. Renal preservation is critical, as bilateral nephrectomy results in immediate renal replacement therapy and subsequent transplantation. However, achieving negative margins is equally important, as margin positivity, typically determined by histopathological assessment, currently mandates adjuvant RT. Radiotherapy, even when conformal, can significantly impair residual renal function and adversely affect adjacent organs, including the bowel and liver. Although there is ongoing debate regarding RT indications based on the histological subtype and the extent of margin involvement, this lies beyond the scope of this review.

In general, outcomes from stage V disease are good [30] from an oncological perspective, but from a renal-function point of view, there is an accelerated decline in the estimated and real glomerular filtration rate (eGFR) [28,31,32,33,34], with a proportion progressing to chronic kidney disease and, in some cases, requiring renal replacement therapy in adulthood [35].

To address the two-fold aim of surgery for NSS, surgeons need to know where the margins of the tumour are intra-operatively. However, there is no standard foolproof way of telling where the margins lie, and current techniques using 3D modelling and/or intra-operative ultrasound are not perfect [36]. In 2022, Abdelhafeez and Davidoff [13] reported on the use of Indocyanine Green (ICG) in NSS and described an inverse relationship between ICG and tumours. Renal parenchyma was found to take up ICG, whereas tumours did not, allowing for macroscopic visualization of the border between a renal tumour and renal parenchyma during NSS.

Further to this work, in 2025, Pachl et al. reported [10] an ex vivo prospective study looking at the macroscopic appearance of a newly resected kidney and tumour following an intra-arterial injection of ICG. This was accompanied by assessment of the pathological slides (formalin-fixed and paraffin-embedded (FFPE)) under a fluorescence-capable microscope. This study confirmed the 2022 observations [13] from St. Jude in that there was an inverse relationship between the renal parenchyma (hyperfluorescent) and the renal tumour (afluorescent) in SIOP low- and intermediate-risk disease. However, high-risk blastemal disease was hyperfluorescent, with no obvious margin between the tumour and the renal parenchyma (Figure 2). Therefore, given this disparity and the fact that, pre-operatively, the histology is unknown, ICG would appear to be an unsuitable dye to use to guide NSS in WTs.

This pattern contrasts with the enhanced permeability and retention effect observed in many adult malignancies and may reflect unique vascular or cellular characteristics of WTs [37,38,39,40].

From a lymph-node perspective, accurate sampling is essential for the staging of WTs and to guide adjuvant therapy. Nodal involvement upstages disease and often necessitates intensification of chemotherapy and/or radiotherapy [41,42]. For WTs, evidence suggests that the likelihood of detecting nodal metastases increases significantly when seven or more nodes are sampled [43]. Furthermore, insufficient sampling has significant effects on the risk of recurrence and survival [44], with data showing that 5-year survival is decreased by up to 14% in patients with WTs where no nodes are sampled. In 1999, Shamberger et al. reported on the National Wilms Study 4 and identified a six-fold increase in local recurrence in stage I patients who had undergone no nodal sampling [41].

Therefore, lymph-node sampling is central to operative and postoperative management of paediatric renal tumours. The Société Internationale d’Oncologie Pédiatrique (SIOP) UMBRELLA protocol recommends the sampling of a minimum of seven nodes for accurate staging [1], and the COG renal tumour guidance suggests a minimum of five sampled nodes [32].

Despite these guidelines, adequate nodal sampling is frequently not achieved, with recommended targets missed in up to 42% of cases [41]. Although lymph-node sampling is recommended rather than formal retroperitoneal lymph-node dissection to minimize morbidity, even targeted sampling can be technically challenging [45].

Increasingly, tumour nephrectomies are being performed via minimally invasive surgery (MIS) for shorter hospital stays, improved cosmesis, and fewer complications [46]. However, MIS’s reduced tactile feedback and limited visualization can amplify challenges and may contribute to lower nodal yields compared with open surgery [14,46,47,48,49]. However, even in open surgery, nodal yields are poor, as alluded to above [48].

Fluorescence-guided lymphatic mapping using ICG has shown promise in adult oncology, demonstrating higher sentinel-node detection rates than blue dye, with an excellent safety profile [50,51]. Early paediatric experience suggests that ICG-guided lymph-node mapping may improve nodal yield in renal tumour surgery, including MIS [52,53] A recent comparative series by Roberts and Pachl [14] reported significantly higher numbers of nodes retrieved in patients receiving ICG, although larger randomised studies are required to confirm these findings (Figure 3).

The results of the GLO-Surgery randomised, controlled trial represent a crucial step toward defining the role of ICG in paediatric oncological surgery and are anticipated to inform future practice (GLO-Surgery ISRCTN26150156).

### 4.1. Future Perspectives

FGS in paediatric renal surgery could employ either non-targeted metabolic or accumulation-based agents (e.g., 5-ALA-induced protoporphyrin IX fluorescence) or targeted probes, including antibody-, peptide-, or small molecule-conjugated fluorophores. Its application in children presents specific challenges, including small operative fields, age-dependent tumour biology, dosing, and safety constraints, as well as the imperative to preserve renal parenchyma. In minimally invasive surgery, where reduced tactile feedback may compromise tumour delineation and lymph-node yield, FGS can partially offset these limitations by improving visual discrimination. However, its utility is constrained by optical limitations such as limited tissue penetration of near-infrared fluorophores and background autofluorescence, with paediatric renal tissue potentially exhibiting optical properties distinct from those of adults.

ICG remains the most widely used dye due to its low cost and exceptionally good safety profile. However, ICG is not a tumour-specific dye, nor is it ideal for tumour resection surgery. This is due to its poor depth penetration and short half-life. Given the breadth of paediatric tumours, it is perhaps unsurprising that some tumours do not fluoresce at all, whilst some fluoresce sometimes and the paediatric surgical oncological community has not yet worked out why NIRF with ICG is sometimes of use and sometimes not.

The development of tumour-specific fluorescent probes represents a critical next step and gold standard to aim for. Agents targeting molecular markers expressed in paediatric renal tumours could significantly improve specificity and margin detection. Although such probes are currently under investigation in adult oncology and preclinical paediatric models, clinical translation remains limited by regulatory and ethical challenges.

Tumour-specific, receptor-targeted fluorophores link an antibody/peptide to a dye, so unbound dye is cleared, while tumour tissue (expressing the target) retains fluorescence. Regulatory agencies treat each antibody dye conjugate as a separate drug, so development timelines and trial requirements are substantial.

However, in principle, this is a viable process, and some drugs have moved from bench to bedside in 10 to 15 years.

The ability to attach to specific cell markers is useful in WTs because they commonly contains multiple cell lineages with distinct surface markers. Surgery is performed before or after neoadjuvant therapy, and biopsy may not reflect predominant intra-tumoural cell types. Therefore, a clinically useful WT-specific FGS agent may need to target one or several surface markers to capture intra- and inter-tumoral heterogeneity.

### 4.2. Promising Targets for Paediatric Renal Tumours

**Glypican3 (GPC3)** is a membrane-associated proteoglycan [54,55,56,57] and B7 Homolog 3 (B7H3) [58] transmembrane protein. Both targets are expressed on Wilms and other paediatric tumours as hepatocellular carcinomas.

GPC3 is encoded by the GPC3 gene, which, in humans, is located at Xq26. This encodes a 580-amino-acid-long protein. GPC3 is noted to regulate Wnt/β-catenin, and its core protein may serve as a co-receptor for Wnt [59,60,61,62,63]. This is a well-known pathway within WT development and may go some way to explaining its expression [64,65,66,67,68].

Its strengths include membrane localization and high specificity where expressed, while its limitations include heterogeneous expression across renal tumour subtypes, with preoperative molecular selection likely needed.

**B7H3** is a 316-amino-acid-long type 1 transmembrane protein that, in humans, is encoded in chromosome 15q24. In cancer tissues, it is an immune checkpoint inhibitor that variably inhibits tumour antigen-specific immune responses and promotes migration, invasion, angiogenesis, chemoresistance, epithelial–mesenchymal transition, and tumour cell metabolism [69].

Its strengths include its often high tumour expression and potential to detect nodal/microscopic disease, while its limitations include variable normal tissue expression and paediatric dosing/safety considerations.

Other potential markers exist within Wilms tumours [70]. Wilms tumour gene 1 (WT1) is the most obvious, but targeting the gene product requires a conjugated dye that will pass intracellularly. Additionally, not all WTs express or overexpress this product, and its immunogenicity is poor [71,72], with peptides being difficult to develop in significant quantities due to their current reliance on CD4+ T cells [73]. Therefore, the difficulties in using WT1 to engender fluorescence would appear to be too great in the current arena.

**Insulin-like growth factor 2 (ILGF 2)** encodes an embryonal growth factor but is unlikely to be an isolated gene product; thus, specific fluorescence for Wilms would be inadequate.

**SIX1/SIX2 gene products** are associated with blastemal-only disease and would not capture intratumoral heterogeneity [74].

**TRIM28** is a classic tumour suppressor gene associated with epithelial histology [75].

**TP53 and MYCN** represent abnormalities that seem to be limited to the anaplastic subtype [76,77].

A considerable number of possibilities exist regarding fluorescent probes that have the potential for in vivo use. A discussion of all of these is outside the scope of this paper. However, there are only two with current FDA approval: Indocyanine Green (ICG) and Pafolacianine (Cytalux), both of which ae cyanine-based dyes. ICG is a non-specific fluorescent agent, whereas Cytalux is specific to folate β receptors.

Additionally, triazole-n-cyanine dyes have shown promise for fluorescence in the near infrared spectrum in in vitro and ex vivo studies [78,79]. Ullah et al. published an extensive review of possible probes in 2024 [80].

### 4.3. Clinical Trials

Finally, there are several current clinical trials investigating the use of fluorescence in surgery. The International Standard Randomized Controlled Trial Number registry (ISCTRN) lists two currently recruiting trials and one suspended study, all assessing the use and function of ICG for cancer surgery. Only one of these trials, the GLO-Surgery study, is being conducted in children’s oncology (https://doi.org/10.1186/ISRCTN26150156, accessed on 9 December 2025).

In the North American Clinical trials registry (https://clinicaltrials.gov) (accessed on 9 December 2025), 770 trials are listed using the search term “Indocyanine Green”, and 394 are listed under search terms “Indocyanine Green AND Cancer”, with seventy-two of these recruiting. Targets include nodal sampling, tumour margins, bowel perfusion, and anastomotic leak.

Most trials returned for a search of “fluorescence guided surgery” assess ICG. However, other dyes include Sodium Fluorescein; 5-ALA; Pafolacianine; TTP-ICG, an ICG-conjugated probe; Panitumumab-IRDye800; ITGA6; SGM-101, a conjugated probe with specific binding capacity for Carcino Embryonic Antigen; Bevacizumab-IRDye800CW; Tozuleristide, an ICG-conjugated probe; GPC3 using anti-GPC3 conjugated to IRDye800CW; IRDye800CW-nimotuzumab, which targets cells over-expressing the EGFR antibody; and cRGD-ZW800-1, a novel fluorescent peptide.

## 5. Conclusions

Fluorescence-guided surgery represents a compelling adjunct in the evolving surgical management of paediatric renal tumours, particularly as the focus shifts from survival alone toward preservation of renal function and reductions in long-term morbidity. Early experience, largely cantered on indocyanine green, suggests that FGS may enhance intraoperative visualization of renal perfusion, tumour–parenchymal interfaces, and lymphatic drainage, with potential benefits for nephron-sparing surgery and nodal staging. These advantages are especially relevant in bilateral disease and minimally invasive surgery, where conventional visual and tactile cues are limited.

## Figures and Tables

**Table 1:** Papers included in the scoping review (WT: Wilms Tumour; ICG: Indocyanine Green; NIRF: Near-InfraRed Fluorescence).

Author	Year	Study Type (Level of Evidence)	Patients with WT (Renal Units)	Patients Receiving Intraparenchymal or Perihilar ICG (Number of Patients with ICG NIRF-Positive Nodes)	Patients Receiving Intravascular ICG for Margin Assessment (Number in Whom ICG NIRF Showed Tumour Margins)
Pachl et al. [10]	2025	Case series (4)	9 (9)	NA	9 (7)
Feng et al. [11]	2023	Case series (4)	4 (4)	3 (3)	4 (4)
Abdelhafeez et al. [12]	2022	Case series (4)	8	8 (8)	NA
Abdelhafeez et al. [13]	2022	Case series (4)	8 (12)	0	8 (8)
Roberts et al. [14]	2024	Case–control (3)	25	7(7)	NA

NA: Not Appicable.

**Table 2:** Current fluorophores.

Fluorophore	Mechanism of Fluorescence	Current Clinical Use	Strengths	Limitations/Considerations
5-Aminolevulinic acid (5-ALA)/Protoporphyrin IX (PpIX)	Oral 5-ALA is metabolized intracellularly to PpIX, which accumulates in tumour cells	Established in adult high-grade glioma surgery; safety and feasibility demonstrated in paediatric brain tumours	Enables metabolic contrast; proven margin delineation in selected tumours; oral administration	Tumour-dependent uptake; limited transferability across tumour types; renal tumour applications remain investigational
Pafolacianine (Cytalux)	Folate receptor α-targeted small-molecule fluorophore with selective tumour binding	Approved for ovarian cancer surgery; investigated in pulmonary metastectomy in young adults	Tumour-specific targeting; exemplifies receptor-targeted fluorophores	Limited paediatric data; efficacy dependent on receptor expression; not yet established in renal tumours
Indocyanine Green (ICG)	Passive vascular and interstitial distribution; fluorescence under near-infrared light	Widely used for perfusion assessment and lymphatic mapping; most commonly used fluorophore in paediatrics	Excellent safety profile; low cost; widely available; established paediatric use	Non-specific; not cell-permeable; short half-life; limited tissue penetration; inconsistent tumour fluorescence

## Data Availability

No new data were created or analyzed in this study. Data sharing is not applicable to this article.

## Supplementary Materials

The following supporting information can be downloaded at: https://www.mdpi.com/article/10.3390/cancers18061041/s1, File S1: Preferred Reporting Items for Systematic reviews and Meta-Analyses extension for Scoping Reviews (PRISMA-ScR) Checklist. Reference [81] is cited in the Supplementary Materials.

## Abbreviations

The following abbreviations are used in this manuscript:

SIOP	International Society of Pediatric Oncology
SIOP-RTSG	SIOP-Renal Tumor Study Group
COG	Children’s Oncology Group
ICG	Indo-cyanine Green
NIRF	Near Infrared Fluorescence
WTs	Wilms Tumours

## References

[1] Vujanić G.M., Gessler M., Ooms A.H.A.G., Collini P., Coulomb-L’Hermine A., D’Hooghe E., De Krijger R.R., Perotti D., Pritchard-Jones K., Vokuhl C. (2018). The UMBRELLA SIOP–RTSG 2016 Wilms tumour pathology and molecular biology protocol. Nat. Rev. Urol..

[2] Troyan S.L., Kianzad V., Gibbs-Strauss S.L., Gioux S., Matsui A., Oketokoun R., Ngo L., Khamene A., Azar F., Frangioni J.V. (2009). The FLARE™ Intraoperative Near-Infrared Fluorescence Imaging System: A First-in-Human Clinical Trial in Breast Cancer Sentinel Lymph Node Mapping. Ann. Surg. Oncol..

[3] Jiang J.X., Keating J.J., Jesus E.M., Judy R.P., Madajewski B., Venegas O., Okusanya O.T., Singhal S. (2015). Optimization of the enhanced permeability and retention effect for near-infrared imaging of solid tumors with indocyanine green. Am. J. Nucl. Med. Mol. Imaging.

[4] Roh C.K., Choi S., Seo W.J., Cho M., Son T., Kim H.I., Hyung W.J. (2020). Indocyanine green fluorescence lymphography during gastrectomy after initial endoscopic submucosal dissection for early gastric cancer. Br. J. Surg..

[5] Dodd A.C., Wadhwani N.R., Lehane A., Brown R., MacQuarrie K.L., Goldstein S.D., Lautz T.B. (2025). Widespread folate receptor expression in pediatric and adolescent solid tumors—Opportunity for intraoperative visualization with the novel fluorescent agent pafolacianine. Oncotarget.

[6] Lehane A., Polites S.F., Dodd A., Goldstein S.D., Lautz T.B. (2024). Let it glow: Intraoperative visualization of pulmonary metastases using pafolacianine, a next-generation fluorescent agent, for young adults undergoing pulmonary metastasectomy. Pediatr. Blood Cancer.

[7] Wellens L.M., Deken M.M., Sier C.F.M., Johnson H.R., De La Jara Ortiz F., Bhairosingh S.S., Houvast R.D., Kholosy W.M., Baart V.M., Pieters A.M.M.J. (2020). Anti-GD2-IRDye800CW as a targeted probe for fluorescence-guided surgery in neuroblastoma. Sci. Rep..

[8] Zhao J., Nakajima K., Ueki M., Terashita Y., Hirabayashi S., Cho Y., Ogawa M., Manabe A. (2025). Near-infrared Photoimmunotherapy using antiGD2 antibody for neuroblastoma and osteosarcoma. EJC Paediatr. Oncol..

[9] Cresswell G.D., Apps J.R., Chagtai T., Mifsud B., Bentley C.C., Maschietto M., Popov S.D., Weeks M.E., Olsen Ø.E., Sebire N.J. (2016). Intra-Tumor Genetic Heterogeneity in Wilms Tumor: Clonal Evolution and Clinical Implications. EBioMedicine.

[10] Pachl M., Bowen C. (2025). Does Indocyanine Green define margins in Wilms tumour? A novel macroscopic and microscopic ex-vivo study. Surg. Oncol..

[11] Feng J., Yang W., Qin H., Xu J., Liu S., Han J., Li N., He L., Wang H. (2023). Clinical application of indocyanine green fluorescence imaging navigation for pediatric renal cancer. Front. Pediatr..

[12] Abdelhafeez A.H., Davidoff A.M., Murphy A.J., Arul G.S., Pachl M.J. (2022). Fluorescence-guided lymph node sampling is feasible during up-front or delayed nephrectomy for Wilms tumor. J. Pediatr. Surg..

[13] Abdelhafeez A.H., Murphy A.J., Brennan R., Santiago T.C., Lu Z., Krasin M.J., Bissler J.J., Gleason J.M., Davidoff A.M. (2022). Indocyanine green-guided nephron-sparing surgery for pediatric renal tumors. J. Pediatr. Surg..

[14] Roberts R., Pachl M. (2024). Intraparenchymal Indocyanine Green Use Improves Nodal Yield During Minimally Invasive Tumor Nephrectomy in Children. J. Laparoendosc. Adv. Surg. Tech. A.

[15] Lakowicz J. (2006). Principles of Fluorescence Spectroscopy.

[16] Valeur B., Berberan-Santos M.N. (2012). Molecular Fluorescence: Principles and Applications.

[17] Weissleder R., Ntziachristos V. (2003). Shedding light onto live molecular targets. Nat. Med..

[18] Vahrmeijer A.L., Hutteman M., van der Vorst J.R., van de Velde C.J., Frangioni J.V. (2013). Image-guided cancer surgery using near-infrared fluorescence. Nat. Rev. Clin. Oncol..

[19] Wang C., Yu Y., Wang Y., Yu J., Zhang C. (2024). Utility and Safety of 5-ALA Guided Surgery in Pediatric Brain Tumors: A Systematic Review. Cancers.

[20] Reynolds J.S., Troy T.L., Mayer R.H., Thompson A.B., Waters D.J., Cornell K.K., Snyder P.W., Sevick-Muraca E.M. (1999). Imaging of spontaneous canine mammary tumors using fluorescent contrast agents. Photochem. Photobiol..

[21] Ito N., Fukuta M., Tokushima T., Nakai K., Ohgi S. (2004). Sentinel node navigation surgery using indocyanine green in patients with lung cancer. Surg. Today.

[22] Tobis S., Knopf J., Silvers C., Yao J., Rashid H., Wu G., Golijanin D. (2011). Near infrared fluorescence imaging with robotic assisted laparoscopic partial nephrectomy: Initial clinical experience for renal cortical tumors. J. Urol..

[23] Joffe B.I., Li G., Gorroochurn P., DeCastro G.J., Lenis A.T., McKiernan J.M., Anderson C.B. (2025). The impact of indocyanine green on partial nephrectomy perioperative outcomes. J. Robot. Surg..

[24] Kuusk T., Brouwer O., Graafland N., Hendricksen K., Donswijk M., Bex A. (2019). Sentinel Lymph Node Biopsy in Renal Tumors: Surgical Technique and Safety. Urology.

[25] Kuusk T., De Bruijn R., Brouwer O.R., De Jong J., Donswijk M., Grivas N., Hendricksen K., Horenblas S., Prevoo W., Valdes Olmos R.A. (2018). Lymphatic Drainage from Renal Tumors In Vivo: A Prospective Sentinel Node Study Using SPECT/CT Imaging. J. Urol..

[26] Kuusk T., Donswijk M.L., Valdes Olmos R.A., De Bruijn R.E., Brouwer O.R., Hendricksen K., Horenblas S., Jozwiak K., Prevoo W., Van Der Poel H.G. (2018). An analysis of SPECT/CT non-visualization of sentinel lymph nodes in renal tumors. EJNMMI Res..

[27] Dome J.S., Fernandez C.V., Mullen E.A., Kalapurakal J.A., Geller J.I., Huff V., Gratias E.J., Dix D.B., Ehrlich P.F., Khanna G. (2013). Children’s Oncology Group’s 2013 blueprint for research: Renal tumors. Pediatr. Blood Cancer.

[28] Fuchs J., Szavay P., Seitz G., Handgretinger R., Schafer J.F., Warmann S.W. (2011). Nephron sparing surgery for synchronous bilateral nephroblastoma involving the renal hilus. J. Urol..

[29] Aldrink J.H., Cost N.G., McLeod D.J., Bates D.G., Stanek J.R., Smith E.A., Ehrlich P.F. (2018). Technical Considerations for Nephron-Sparing Surgery in Children: What is Needed to Preserve Renal Units?. J. Surg. Res..

[30] Murphy A.J. (2024). Pursuit of the optimal therapeutic approach and intensity for children with bilateral Wilms tumour. Br. J. Cancer.

[31] Murphy A.J., Brzezinski J., Renfro L.A., Tornwall B., Malek M.M., Benedetti D.J., Cost N.G., Smith E.A., Aldrink J., Romao R.L.P. (2024). Long-term outcomes and patterns of relapse in patients with bilateral Wilms tumor or bilaterally predisposed unilateral Wilms tumor, a report from the COG AREN0534 study. Int. J. Cancer.

[32] Dome J.S., Graf N., Geller J.I., Fernandez C.V., Mullen E.A., Spreafico F., Van den Heuvel-Eibrink M., Pritchard-Jones K. (2015). Advances in Wilms Tumor Treatment and Biology: Progress Through International Collaboration. J. Clin. Oncol..

[33] Perotti D., Williams R.D., Wegert J., Brzezinski J., Maschietto M., Ciceri S., Gisselsson D., Gadd S., Walz A.L., Furtwaengler R. (2023). Hallmark discoveries in the biology of Wilms tumour. Nat. Rev. Urol..

[34] Romao R.L.P., Aldrink J.H., Renfro L.A., Mullen E.A., Murphy A.J., Brzezinski J., Malek M.M., Benedetti D.J., Cost N.G., Smith E. (2024). Bilateral Wilms tumor with anaplasia: A report from the Children’s Oncology Group Study AREN0534. Pediatr. Blood Cancer.

[35] Ceccanti S., Cozzi F., Cervellone A., Zani A., Cozzi D.A. (2019). Volume and function of the operated kidney after nephron-sparing surgery for unilateral renal tumor. J. Pediatr. Surg..

[36] Chaussy Y., Vieille L., Lacroix E., Lenoir M., Marie F., Corbat L., Henriet J., Auber F. (2020). 3D reconstruction of Wilms’ tumor and kidneys in children: Variability, usefulness and constraints. J. Pediatr. Urol..

[37] Abdelhafeez A., Talbot L., Murphy A.J., Davidoff A.M. (2021). Indocyanine Green-Guided Pediatric Tumor Resection: Approach, Utility, and Challenges. Front. Pediatr..

[38] Maeda H. (2015). Toward a full understanding of the EPR effect in primary and metastatic tumors as well as issues related to its heterogeneity. Adv. Drug Deliv. Rev..

[39] Maeda H., Wu J., Sawa T., Matsumura Y., Hori K. (2000). Tumor vascular permeability and the EPR effect in macromolecular therapeutics: A review. J. Control. Releas.

[40] Sun R., Xiang J., Zhou Q., Piao Y., Tang J., Shao S., Zhou Z., Bae Y.H., Shen Y. (2022). The tumor EPR effect for cancer drug delivery: Current status, limitations, and alternatives. Adv. Drug Deliv. Rev..

[41] Shamberger R.C., Guthrie K.A., Ritchey M.L., Haase G.M., Takashima J., Beckwith J.B., D’Angio G.J., Green D.M., Breslow N.E. (1999). Surgery-Related Factors and Local Recurrence of Wilms Tumor in National Wilms Tumor Study 4. Ann. Surg..

[42] Ehrlich P.F., Anderson J.R., Ritchey M.L., Dome J.S., Green D.M., Grundy P.E., Perlman E.J., Kalapurakal J.A., Breslow N.E., Shamberger R.C. (2013). Clinicopathologic findings predictive of relapse in children with stage III favorable-histology Wilms tumor. J. Clin. Oncol..

[43] Kieran K., Anderson J.R., Dome J.S., Ehrlich P.F., Ritchey M.L., Shamberger R.C., Perlman E.J., Green D.M., Davidoff A.M. (2012). Lymph node involvement in Wilms tumor: Results from National Wilms Tumor Studies 4 and 5. J. Pediatr. Surg..

[44] Raval M.V., Bilimoria K.Y., Bentrem D.J., Stewart A.K., Winchester D.P., Ko C.Y., Reynolds M. (2010). Nodal Evaluation in Wilms’ Tumors: Analysis of the National Cancer Data Base. Ann. Surg..

[45] Ziogas I.A., Khomiak A., Olson K.E., Moris D.P., Robbins A.J., Stevens J., Acker S.N., Roach J.P., Corkum K.S., Cost N.G. (2025). The Impact of Lymph Node Ratio for Children with Wilms Tumors: A National Cancer Database Analysis. Cancers.

[46] Mentessidou A., Djendov F., Long A.M., Jackson C. (2024). Systematic Review and Meta-analysis of Laparoscopic Versus Open Radical Nephrectomy for Paediatric Renal Tumors with Focus on Wilms’ Tumor. Ann. Surg..

[47] Warmann S.W., Godzinski J., van Tinteren H., Heij H., Powis M., Sandstedt B., Graf N., Fuchs J. (2014). Minimally invasive nephrectomy for Wilms tumors in children—Data from SIOP 2001. J. Pediatr. Surg..

[48] Gavens E., Arul G.S., Pachl M. (2020). A single centre matched pair series comparing minimally invasive and open surgery for the resection of pediatric renal tumours. Surg. Oncol..

[49] van der Merwe E., Pachl M. (2022). Minimally invasive surgery (MIS) for paediatric renal tumours: A narrative review of the technical aspects. Laparosc. Surg..

[50] Cahill R.A., Anderson M., Wang L.M., Lindsey I., Cunningham C., Mortensen N.J. (2012). Near-infrared (NIR) laparoscopy for intraoperative lymphatic road-mapping and sentinel node identification during definitive surgical resection of early-stage colorectal neoplasia. Surg. Endosc..

[51] Rijs Z., Jeremiasse B., Shifai N., Gelderblom H., Sier C.F.M., Vahrmeijer A.L., van Leeuwen F.W.B., van der Steeg A.F.W., van de Sande M.A.J. (2021). Introducing Fluorescence-Guided Surgery for Pediatric Ewing, Osteo-, and Rhabdomyosarcomas: A Literature Review. Biomedicines.

[52] Harris A.C., Choudhury S., Pachl M. (2023). Early results of minimally invasive fluorescent guided pediatric oncology surgery with delivery of indocyanine green during induction of anesthesia. Photodiagnosis Photodyn. Ther..

[53] Pachl M.J. (2021). Fluorescent Guided Lymph Node Harvest in Laparoscopic Wilms Nephroureterectomy. Urology.

[54] Toretsky J.A., Zitomersky N.L., Eskenazi A.E., Voigt R.W., Strauch E.D., Sun C.C., Huber R., Meltzer S.J., Schlessinger D. (2001). Glypican-3 Expression in Wilms Tumor and Hepatoblastoma. J. Pediatr. Hematol. Oncol..

[55] Kinoshita Y., Tanaka S., Souzaki R., Miyoshi K., Kohashi K., Oda Y., Nakatsura T., Taguchi T. (2015). Glypican 3 Expression in Pediatric Malignant Solid Tumors. Eur. J. Pediatr. Surg..

[56] Saikali Z., Sinnett D. (2000). Expression of glypican 3 (GPC3) in embryonal tumors. Int. J. Cancer.

[57] Tretiakova M., Zynger D.L., Luan C., Andeen N.K., Finn L.S., Kocherginsky M., Teh B.T., Yang X.J. (2015). Glypican 3 overexpression in primary and metastatic Wilms tumors. Virchows Arch..

[58] Agarwal S., Fang L., McGowen K., Yin J., Bowman J., Ku A.T., Alilin A.N., Corey E., Roudier M.P., True L.D. (2023). Tumor-derived biomarkers predict efficacy of B7H3 antibody-drug conjugate treatment in metastatic prostate cancer models. J. Clin. Investig..

[59] Gao W., Kim H., Feng M., Phung Y., Xavier C.P., Rubin J.S., Ho M. (2014). Inactivation of Wnt signaling by a human antibody that recognizes the heparan sulfate chains of glypican-3 for liver cancer therapy. Hepatology.

[60] Gao W., Xu Y., Liu J., Ho M. (2016). Epitope mapping by a Wnt-blocking antibody: Evidence of the Wnt binding domain in heparan sulfate. Sci. Rep..

[61] Kolluri A., Ho M. (2019). The Role of Glypican-3 in Regulating Wnt, YAP, and Hedgehog in Liver Cancer. Front. Oncol..

[62] Wang D., Gao Y., Zhang Y., Wang L., Chen G. (2019). Glypican-3 promotes cell proliferation and tumorigenesis through up-regulation of beta-catenin expression in lung squamous cell carcinoma. Biosci. Rep..

[63] Ortiz M.V., Roberts S.S., Glade Bender J., Shukla N., Wexler L.H. (2019). Immunotherapeutic Targeting of GPC3 in Pediatric Solid Embryonal Tumors. Front. Oncol..

[64] Coorens T.H.H., Treger T.D., Al-Saadi R., Moore L., Tran M.G.B., Mitchell T.J., Tugnait S., Thevanesan C., Young M.D., Oliver T.R.W. (2019). Embryonal precursors of Wilms tumor. Science.

[65] Treger T.D., Chowdhury T., Pritchard-Jones K., Behjati S. (2019). The genetic changes of Wilms tumour. Nat. Rev. Nephrol..

[66] Treger T.D., Wegert J., Wenger A., Coorens T.H.H., Al-Saadi R., Kemps P.G., Kennedy J., Parks C., Anderson N.D., Hodder A. (2025). Predisposition Footprints in the Somatic Genome of Wilms Tumors. Cancer Discov..

[67] Young M.D., Mitchell T.J., Custers L., Margaritis T., Morales-Rodriguez F., Kwakwa K., Khabirova E., Kildisiute G., Oliver T.R.W., de Krijger R.R. (2021). Single cell derived mRNA signals across human kidney tumors. Nat. Commun..

[68] Young M.D., Mitchell T.J., Vieira Braga F.A., Tran M.G.B., Stewart B.J., Ferdinand J.R., Collord G., Botting R.A., Popescu D.-M., Loudon K.W. (2018). Single-cell transcriptomes from human kidneys reveal the cellular identity of renal tumors. Science.

[69] Kontos F., Michelakos T., Kurokawa T., Sadagopan A., Schwab J.H., Ferrone C.R., Ferrone S. (2021). B7-H3: An Attractive Target for Antibody-based Immunotherapy. Clin. Cancer Res..

[70] Zheng H., Liu J., Pan X., Cui X. (2023). Biomarkers for patients with Wilms tumor: A review. Front. Oncol..

[71] Sawada A., Inoue M., Kondo O., Yamada-Nakata K., Ishihara T., Kuwae Y., Nishikawa M., Ammori Y., Tsuboi A., Oji Y. (2016). Feasibility of Cancer Immunotherapy with WT1 Peptide Vaccination for Solid and Hematological Malignancies in Children. Pediatr. Blood Cancer.

[72] Nian Q., Lin Y., Zeng J., Zhang Y., Liu R. (2025). Multifaceted functions of the Wilms tumor 1 protein: From its expression in various malignancies to targeted therapy. Transl. Oncol..

[73] Krishnadas D.K., Stamer M.M., Dunham K., Bao L., Lucas K.G. (2011). Wilms’ tumor 1-specific cytotoxic T lymphocytes can be expanded from adult donors and cord blood. Leuk. Res..

[74] Wegert J., Ishaque N., Vardapour R., Geörg C., Gu Z., Bieg M., Ziegler B., Bausenwein S., Nourkami N., Ludwig N. (2015). Mutations in the SIX1/2 pathway and the DROSHA/DGCR8 miRNA microprocessor complex underlie high-risk blastemal type Wilms tumors. Cancer Cell.

[75] Hol J.A., Diets I.J., De Krijger R.R., Van Den Heuvel-Eibrink M.M., Jongmans M.C., Kuiper R.P. (2021). TRIM28 variants and Wilms’tumour predisposition. J. Pathol..

[76] Williams R.D., Chagtai T., Alcaide-German M., Apps J., Wegert J., Popov S., Vujanic G., Van Tinteren H., Van Den Heuvel-Eibrink M.M., Kool M. (2015). Multiple mechanisms of MYCN dysregulation in Wilms tumour. Oncotarget.

[77] Ooms A.H.A.G., Gadd S., Gerhard D.S., Smith M.A., Guidry Auvil J.M., Meerzaman D., Chen Q.-R., Hsu C.H., Yan C., Nguyen C. (2016). Significance of TP53 Mutation in Wilms Tumors with Diffuse Anaplasia: A Report from the Children’s Oncology Group. Clin. Cancer Res..

[78] Mellanby R.J., Scott J.I., Mair I., Fernandez A., Saul L., Arlt J., Moral M., Vendrell M. (2018). Tricarbocyanine N-triazoles: The scaffold-of-choice for long-term near-infrared imaging of immune cells in vivo. Chem. Sci..

[79] Thomas C.N., Alfahad N., Capewell N., Cowley J., Hickman E., Fernandez A., Harrison N., Qureshi O.S., Bennett N., Barnes N.M. (2022). Triazole-derivatized near-infrared cyanine dyes enable local functional fluorescent imaging of ocular inflammation. Biosens. Bioelectron..

[80] Ullah Z., Roy S., Gu J., Ko Soe S., Jin J., Guo B. (2024). NIR-II Fluorescent Probes for Fluorescence-Imaging-Guided Tumor Surgery. Biosensors.

[81] Tricco A.C., Lillie E., Zarin W., O’Brien K.K., Colquhoun H., Levac D., Moher D., Peters M.D., Horsley T., Weeks L. (2018). PRISMA Extension for Scoping Reviews (PRISMA-ScR): Checklist and Explanation. Ann. Intern. Med..

